# Surgery vs. Biopsy in the Treatment of Butterfly Glioblastoma: A Systematic Review and Meta-Analysis

**DOI:** 10.3390/cancers14020314

**Published:** 2022-01-09

**Authors:** Shreya Chawla, Vasileios K. Kavouridis, Alessandro Boaro, Rasika Korde, Sofia Amaral Medeiros, Heba Edrees, Elisabetta Mezzalira, Francesco Sala, Rania A. Mekary, Timothy R. Smith

**Affiliations:** 1 Computational Neuroscience Outcomes Center, Department of Neurosurgery, Brigham and Women’s Hospital, Boston, MA 02115, USA; shreya.chawla@kcl.ac.uk (S.C.); vkavouridis@me.com (V.K.K.); Elisabetta.mezzalira@univr.it (E.M.); rania.mekary@mcphs.edu (R.A.M.); trsmith@bwh.harvard.edu (T.R.S.); 2 Faculty of Life Sciences and Medicine, King’s College London, London WC2R 2LS, UK; 3 Department of Neurosurgery, St. Olavs Hospital, 7030 Trondheim, Norway; 4 Section of Neurosurgery, Department of Neuroscience, Biomedicine and Movement Sciences, Institute of Neurosurgery, University of Verona, 37129 Verona, Italy; francesco.sala@univr.it; 5 School of Pharmacy, MCPHS University, Boston, MA 02215, USA; rkord1@stu.mcphs.edu (R.K.); hh.edrees@gmail.com (H.E.); 6 Faculdade de Medicina da Universidade de São Paulo, São Paulo 01246903, Brazil; sofia.amaral@fm.usp.br; 7 Department of Diagnostic and Public Health, University of Verona, 37129 Verona, Italy

**Keywords:** butterfly glioblastoma, prognosis, meta-analysis

## Abstract

**Simple Summary:**

There is uncertainty regarding the role of surgical resection in the management of butterfly glioblastoma (bGBM). We therefore investigated this question by pooling available data from the literature and performing a systematic review and meta-analysis. Our results show that operative management of bGBM was associated with longer overall survival compared with biopsy alone. This effect persisted in both >80% and <80% extent of resection subgroups. At the same time, complications were not statistically significantly higher; however, these were numerically larger for surgery. Our study corroborates findings from smaller studies and supports the consideration of surgery in the treatment of bGBM patients.

**Abstract:**

Butterfly glioblastomas (bGBM) are grade IV gliomas that spread to bilateral hemispheres by infiltrating the corpus callosum. Data on the effect of surgery are limited to small case series. The aim of this meta-analysis was to compare resection vs. biopsy in terms of survival outcomes and postoperative complications. A systematic review of the literature was conducted using PubMed, EMBASE, and Cochrane databases through March 2021 in accordance with the PRISMA checklist. Pooled hazard ratios were calculated and meta-analyzed in a random-effects model including assessment of heterogeneity. Out of 3367 articles, seven studies were included with 293 patients. Surgical resection was significantly associated with longer overall survival (HR 0.39, 95%CI 0.2–0.55) than biopsy. Low heterogeneity was observed (I^2^: 0%). In further analysis, the effect persisted in extent of resection subgroups of both ≥80% and <80%. No statistically significant difference between surgery and biopsy was detected in terms of postoperative complications, although these were numerically larger for surgery. In patients with bGBM, surgical resection was associated with longer survival prospects compared with biopsy.

## 1. Introduction

Butterfly glioblastoma (bGBM) refers to WHO grade IV gliomas that infiltrate the corpus callosum and spread to bilateral cerebral hemispheres. These rare tumors carry an extremely poor prognosis as invasion of the corpus callosum significantly increases the risk of tumor dissemination [1,2,3,4,5,6]. Unsurprisingly, in a paper examining survival in patients with butterfly lesions of varying histology, glioblastoma was found to be the unfavorable predictor of survival [7]. There has been much debate about the optimal management of bGBM patients; while typically considered inoperable and managed with biopsy and chemoradiation, newer reports have advocated for resection in carefully selected cases with the goal of cytoreduction. Given the rarity of bGBM, all available evidence comes from case reports or single institution case series, where the results seem to favor resection over biopsy in terms of overall survival. However, data on post-operative deficits and performance status are usually less systematically analyzed and reported, despite being of critical importance in the decision-making process for these patients. With the progressive refinement of operative and monitoring techniques and with the development of new treatment paradigms, it is highly relevant to provide additional, high quality evidence regarding the effect of current treatment options, in order to provide physicians and families with safer treatment guidance [8]. Thus, we designed a systematic review and meta-analysis with the aim to compare surgical resection and biopsy with regard to (1) overall survival and (2) post-operative deficits in patients diagnosed with bGBM.

## 2. Materials and Methods

A systematic review of the literature was conducted in accordance with the 2009 Preferred Reporting Items for Systematic Reviews and Meta-Analyses (PRISMA) Statement [9]. The review protocol was registered with PROSPERO in September 2020 (registration number CRD42020188147). Systematic adherence to the PRISMA checklist for study search, screening, data extraction, and analysis was carefully implemented.

### 2.1. Search Strategy

MEDLINE (PubMed), Embase, and Cochrane databases were searched using keywords related to glioblastoma, butterfly glioma, surgery and biopsy up to March 2021. Randomized controlled trials and observational studies describing the outcomes of surgical resection vs. biopsy on overall survival or progression-free survival of bGBM were included. Relevant studies identified in the bibliographies of the reviewed papers were also included. Duplicate publications of the same trials, pediatric studies (<18), non-English language papers, reviews, and case reports were excluded. Two independent authors (A.B., S.C.) screened the titles and abstracts of articles against the inclusion and exclusion criteria. Subsequently, full texts were reviewed by 5 independent authors (A.B., H.E., R. K., S.C., V.K.) against eligibility criteria for final selection. Any disagreements between the authors were resolved by discussion.

### 2.2. Data Extraction

A pre-designed excel sheet was used to extract and organize the data into categories by 4 independent authors (A.B., R.K., S.C., S.M.). Extracted data included (1) study information; (2) clinical and treatment characteristics e.g., sample size, age, gender, intervention type (biopsy/surgery), extent of resection (EOR), adjuvant therapy; (3) outcome measures including overall survival (OS), progression-free survival (PFS) (hazard ratios for OS and PFS were recorded where available) and post-operative complications and; (4), study limitations. If quantitative data on relative outcomes were not available, the relevant authors were contacted for further information.

### 2.3. Quality Assessment

Four authors (A.B., R.K., S.C., S.M.) independently assessed the quality of each included study using the questionnaire by Chan and Bhandari [10] for case series. The questionnaire assessed all studies based on whether their study objective, protocol, inclusion and exclusion criteria, time interval, and patient enrollment were well defined and if the studies had prospective collection of outcome data and a high follow-up. Each category had one point associated to it with the highest possible score of 8. The higher the score on the questionnaire was, the better the quality of the study was. Any disagreements were discussed among the authors. As fewer than 10 studies were pooled in our meta-analysis, we were unable to identify the small study effect through funnel plots or through Begg’s and Egger’s tests [11,12].

### 2.4. Statistical Methods and Analysis

Hazard ratios comparing surgical resection to biopsy for bGBM survival outcomes were pooled. Where the studies provided median survival instead of hazard ratios, univariate hazard ratios were derived using the equation in Appendix A. We derived 95% confidence intervals (CIs) for studies reporting HR without 95% CI using the equation in Appendix B [13]. If a study provided a multivariate hazard ratio, it was included in a sensitivity analysis.

The pooled point estimate and its 95% confidence interval was then calculated in the meta-analysis using the DerSimonian and Laird random-effects model [14]. Sub-group analysis was performed based on EOR categories ≥80% and <80%. The analysis was performed in Comprehensive Meta-Analysis version 3. We pooled the complications data using the meta package in R. Unless otherwise specified, a two-sided *p* value of <0.05 was considered statistically significant.

### 2.5. Heterogeneity Assessment and Analysis

Presence of heterogeneity was assessed using Cochrane Q statistic with a significance level of *p* < 0.10. Degree of heterogeneity among studies was determined using the I^2^ value. Degree of heterogeneity was reported to be low, medium and high with I^2^ values of 25%, 50% and 75% respectively [15].

## 3. Results

**Figure 1:**
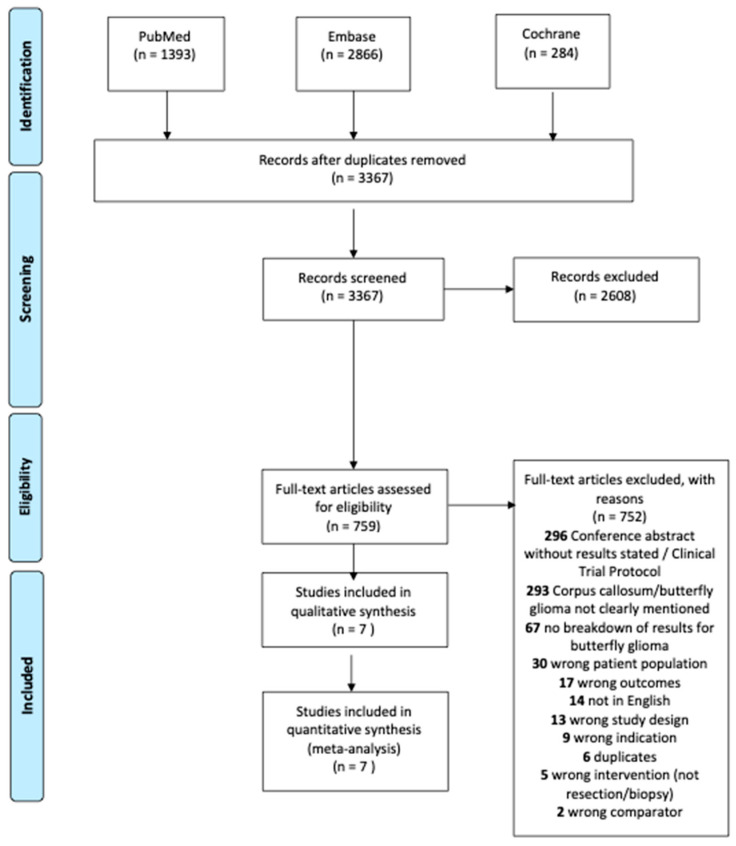
PRISMA flow diagram of included articles.

A total of 3367 studies were identified during the initial search. Of those, 2608 were excluded in title and abstract screening. After full text review, 752 studies were excluded as shown in Figure 1. Seven [2,3,6,16,17,18,19] unique studies were eligible for the meta-analysis including 293 patients with butterfly glioblastomas. Both univariate and multivariate hazard ratios (adjusted for age, Karnofsky Performance Status at diagnosis, and pre-operative tumor volume) were available in one study [6], while univariate hazard ratios were derived from median survival times for six studies [2,3,16,17,18,19].

### 3.1. Characteristics of Included Trials

Six [2,3,6,16,17,18] of the included studies were case series and one [19], which was only available as a conference abstract, followed a case-control design. Study characteristics are presented in Table 1. The studies included a total of 293 patients undergoing either biopsy or resection for confirmed butterfly glioma. Study period varied between five to twelve years and most of the studies were based in the United States (four out of seven).

Further intervention details are specified in Table 2 and Table 3. All studies except for the abstract by Hall et al. [19] reported pre-operative tumor volumes. The mean preoperative tumor volume was 48.9 cm^3^ in the resection group and 44.1 cm^3^ in the biopsy group. The IDH1/2 mutation and MGMT methylation status were present in four studies in 139 and 130 cases respectively. The mean age was 58.3 and 62.8 in the resection and biopsy group, respectively. Information on adjuvant chemotherapy and radiotherapy was provided in four [3,6,16,17] out of the seven studies (Table 3).

**Figure 2:**
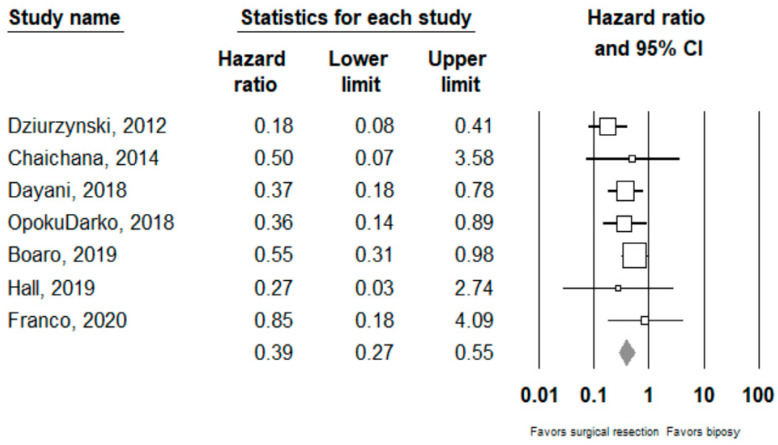
Forest plots showing hazard ratios of surgery vs biopsy for overall survival. Each study is shown by the point estimate of the hazard ratio and 95% confidence intervals (extending lines). The diamond center represents the estimated pooled hazard ratio and the width represent its 95% confidence interval (labelled total).

**Figure 3:**
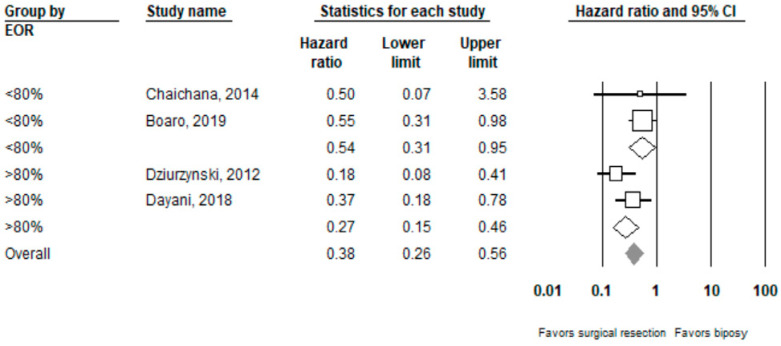
Forest plots showing hazard ratios of surgery vs. biopsy for overall survival stratified by EOR (≥80% vs. <80%).

Performing surgical resection as opposed to biopsy was significantly associated with better overall survival (HR = 0.39, 95% CI 0.27–0.55, *n* = 7 studies; I^2^: 0%, p-heterogeneity *=* 0.44). Further subgroup analysis showed that surgical resection was associated with improved overall survival in both the ≥80% (HR = 0.27, 95% CI 0.15 –0.46, *n* = 2, I^2^: 38.3%) and <80% (HR 0.54, 95% CI 0.31–0.95, *n* =2, I^2^: 0%) EOR subgroups; *p*-value comparing both subgroups: 0.08. Full results can be seen in Figure 2 and Figure 3.

### 3.2. Complications and Clinical Outcomes

In terms of complications, Boaro [17] and Dayani [6] only reported post-operative deficits in the resection group. Conversely, Hall [19] and Franco [16] found no significant difference in the number of complications between the two groups. Opoku-Darko [18] did not do a between-group comparison but found two complications in the resection group compared with one in the biopsy group. A detailed description of the post-operative deficits can be found in Appendix C. After pooling complication rates in clinically meaningful categories and comparing them between the two management groups, we found no statistically significant differences; however, the complications were numerically larger for surgery (Table 4).

### 3.3. Bias

Quality of the studies was assessed using the questionnaire by Chan and Bhandari [10]. The quality score for all studies ranged from 6–7. All studies had a well-defined study objective, a defined protocol, clinically relevant outcomes, clear inclusion/exclusion criteria and a high follow-up rate. Only one study had consecutive patient enrollment, and none had prospective data collection as shown in Appendix D. We were unable to assess the quality of Hall et al. 2019 [19] as the study was only an abstract.

## 4. Discussion

In the current systematic review and meta-analysis, we demonstrated a significantly longer OS with surgical resection of bGBM compared with biopsy only. This advantage remained significant after considering patients with just subtotal (study median EOR < 80%) resection. Taken individually, four studies reported significantly different hazard ratios for OS in favor of resection compared with biopsy, whereas three studies did not reach statistical significance although the direction of the association was consistently in favor of surgery. Our results were similar to a recent meta-analysis by Chojak et al. [20], which showed a decreased six-month mortality rate (RR 0.63) in resected cases; however, they did not find significant differences at the 12- and 18-month intervals. The latter finding is perhaps not surprising given that median OS in surgically treated BG is approximately 12 months, and as such few patients would be still alive at those later intervals to allow for meaningful statistical comparisons. By including two additional studies, resulting in a 34% increase in the studied population, we were able to corroborate these findings, which is of particular importance considering the rarity of the disease. Moreover, by using the 80% EOR cut-off, we were able to provide additional evidence that surgical resection, even if partial, had a distinct survival advantage. It should be noted that similar conclusions were reached in our evaluation of a national cohort of bGBM patients, where gross or subtotal resection were significantly associated with a favorable prognosis [17].

Given the limited life expectancy of bGBM patients, which makes questions about quality of life even more pertinent, it is noteworthy how little attention has been devoted in the literature to examination of functional outcomes after different management approaches including surgery. In light of the aforementioned survival benefits of operative treatment, postoperative status can have critical importance for patient counseling when discussing the risk–benefit ratio between biopsy and resection. While expert opinion historically tended to discourage surgery in patients with bGBM due to high risk of deficit and/or complications, no hard evidence has been provided, even in recent works (Chojak) [20], which could adequately support this claim. We pooled the available evidence and provided here a review of Karnofsky Performance Status (KPS), post-operative complications, and neurological deficits with the goal of providing a basis to include surgical safety and functional outcome into the treatment decision-making process.

Regarding postoperative KPS, two studies presented either no significant difference between biopsy and resection groups (Dziurzynski, OpokuDarko) [3,18], a non-significant difference between pre-operative and post-operative KPS in the resection group (Dayani, Franco) [6,16], or even reported a temporary improvement of KPS at three months post-op in the resection group (Franco) [16]. In relation to complications (hemorrhage, ischemia, thromboembolic events, infections) and neurological deficits (involving either motor, language, vision or cognitive domains), no study was able to detect a difference neither between biopsied and resected BG (Chaichana, OpokuDarko, Hall, Franco) [2,16,18,19], nor between bGBM and regular GBM (Chaichana) [2]. Interestingly, while the immediate post-operative rate of complications or neurological deficits could be as high as 35%, two studies reported a complete resolution rate of 61.5% and 66.6% (Dayani, Boaro) [6,17]. Moreover, in one study (Franco) [16] the authors reported a better neurological outcome at one year for patients undergoing resection, attributing this result to a reduced edema and mass effect due to gross total resection of the lesions. It is also interesting to observe that studies reporting a median resection of >80% presented a similar rate of worsened functional outcomes compared with studies reporting <80% resection (respectively 33.7 and 35%).

In summary, one can claim that surgical resection of bGBMs, which needs not be radical, is associated with longer survival prospects. Our paper also found that surgical resection was not associated with prohibitive rates of postoperative neurological deficits or other complications, which if they occur tend to be transient in nature; however, conclusions should not be overstated out of these findings as these statistical comparisons might be subject to confounding and lack of power.

Even though the included studies supported a more aggressive surgical approach in bGBM patients, only a few delved into the technical aspects of how to go about achieving this. In this regard, Burks et al. [21] provided an interesting perspective in their work. They presented a novel cingulate-sparing technique based on the anatomic-functional study of the cingulum and its connectivity within the default mode network as a way to improve EOR and reduce the occurrence of post-operative abulia in patients with frontal butterfly glioma (both low grade and high grade). They reported an EOR greater than 90% in 87% of patients undergoing a cingulate-sparing technique with only one case of transient abulia, whereas patients undergoing a traditional resection approach reported a 28% rate of abulia at six weeks post-op. Still, this last group of patients presented a 33% resolution of abulia between immediate post-operative and follow-up (44% to 28%), highlighting the presence of a significant recovery potential. The complication rate was lower in the CST group (13% vs. 28%) but no statistical significance was demonstrated (*p* = 0.28) [21]. This work supports the idea that the intimate knowledge of the anatomical structures surrounding and connecting the corpus callosum to the hemispheres combined with the utilization of state-of-the-art neurophysiological monitoring techniques, along with accurate patient selection, could be key elements in making a procedure that has historically been deemed futile into a more established, safe and effective operation.

Some important limitations need to be taken into account in the interpretation of our results. First of all, due to the limited sample size of the included studies, we caution against major conclusions regarding treatment recommendations. The fact that most of these studies were case series meant the HRs were unadjusted (except for one study) [6] and were thus subject to confounding bias. In fact, three [2,18,19] out of the seven studies did not provide information about adjuvant therapy provided to the patients. Moreover, the inability to consider additional and potentially confounding features such as tumor volume, specific types of second lines of chemotherapy or radiotherapy features, and tumor-related mutations and gene expression patterns, precluded us not only from conducting a pooled multivariate-adjusted hazard ratio, but also from using meta-regression methods to account for these sources of heterogeneity. Additionally, only English language papers were included in the search, allowing for potential language bias. Other limitations include the inability to assess the small study effect due to the limited number of studies included. As with all meta-analyses, its strength is contingent on that of the included studies.

On the other hand, this meta-analysis has some important strengths. It is the first meta-analysis to systematically explore not only survival in bGBM patients but also, and probably more importantly, postoperative complications. In regards to the comparison of survival between tumor resection and biopsy groups, this paper represents the most complete analysis of the literature with an initial search of more than 3000 articles and a final patient population 30% larger in comparison with similar works.

## 5. Conclusions

Surgical resection was associated with favorable survival prospects compared to biopsy in bGBM patients. This effect was also apparent in cases with subtotal resection (<80%). Future research should focus on the examination of the molecular background of these tumors and the identification of prognostic factors that can aid in the selection of patients more suitable for operative management. Additionally, better-designed comparative studies are needed that adjust for confounding, and provide additional information, such as receipt of adjuvant therapy, in order to make the resection and biopsy groups as comparable as possible and strengthen findings regarding neurological deficits and postoperative complications.

## Figures and Tables

**Table 1:** Study characteristics of included articles.

Reference	Country	Study Design	Study Period	Sample Size		Male Sex (%)	Mean Age (Years)
				Resection	Biopsy		
Dziurzynski, 2012	USA	Case series	2000–2010	11	12	48%	56.3
Chaichana, 2014	USA	Case series	2007–2012	29	19	52%	NR
Dayani, 2018	USA, Ireland	Case series	2004–2014	14	25	59%	57.8
OpokuDarko, 2018	Canada	Case series	2004–2016	9	20	62%	59.8
Hall, 2019	UK	Case control	2011–2017	37 *		64.9%	62.7 *
Franco, 2020	Germany	Case series	2005–2017	25	30	61.8%	64
Boaro, 2021	USA, Italy	Case series	2008–2018	26	36	51.6%	64.3

* Sample size not stratified by treatment modality.

**Table 2:** Comparison of pre-operative tumor volume, genetic profile, age, and sex between the resection and biopsy groups.

	Resection	Biopsy
Mean pre-operative tumor volume in cm^3^	48.9	44.1
IDH1/2 Mutation% (n/N)	1.59% (1/63)	2.63% (2/76)
MGMT Methylation% (n/N)	34.4% (21/61)	34.8% (24/69)
Mean age *, years	58.3	62.8
Sex (% Male) *	57.3%	65.2%

* Data do not include Dziurynski and Hall as age and sex data were not stratified by intervention group.

**Table 3:** Intervention and outcomes of included studies for all patients (surgery and biopsy).

Reference	Receipt of Adjuvant Therapy (%)		Overall Survival (Median Months)	Extent of Resection (Median)
	Chemotherapy	Radiation		
Dziurzynski, 2012	39.10%	52.20%	5.9	100%
Chaichana, 2014	NR	NR	NR	61.4%
Dayani, 2018	43.60%	12.80%	3.2	83.04%
OpokuDarko, 2018	NR	NR	3.3	NR
Boaro, 2021	71.00%	75.80%	8.7	72.30%
Hall, 2019	NR	NR	3.3	NR
Franco, 2020	52.5%	34.6%	8.3	NR

NR: not reported.

**Table 4:** Pooled incidence of complications (95% confidence intervals) ^†^ comparing surgery to biopsy.

Complication	All	Surgery Subgroup	*N* of Studies	Biopsy Subgroup	*N* of Studies	Surgery vs. Biopsy *p*-Value
Motor	7% (2%, 20%)	11% (4%, 27%)	5	1.3% (0%, 100%)	3	0.33
Speech and Language	8% (4%, 15%)	11% (5%, 22%)	5	4% (0.3%, 37%)	3	0.15
Visuospatial	0.4% (0%, 29%)	1.59% (0.02%, 51.8%)	5	0% (0%, 100%)	3	>0.99
Cognition	0.5% (0.01%, 30%)	2% (0.04%, 49%)	5	0% (0%, 100%)	3	>0.99
Seizures	10% (0.01%, 99%)	NR	NR	NR	NR	NR
Hydrocephalus	7.4% (1.3%, 33%)	7.84% (0.01%, 98%)	2	7% (2%, 23%)	1	0.85
Total	21% (14%, 31%)	28% (18–41%)	5	13% (4%, 37%)	3	0.01

NR: not reported.

## Appendix A

Hazard ratio HR = Median 2/Median 1.

## Appendix B

1. calculate the test statistic for a normal distribution test, *z*, from P3: *z* = −0.862 + √[0.743 − 2.404 × log(P)].
2. Est = log(HR).
3. calculate the standard error: *SE* = − *Est*/*z* (ignoring minus signs).
4. calculate the 95% CI: *Est* − 1.96 × *SE* to *Est* + 1.96 × *SE*.
5. Convert to natural scale.

## Appendix C

**Table A1:** Post-operative complications compared by group.

Reference	Resection	Biopsy
Dziurzynski, 2012	NR	NR
Chaichana, 2014	Motor deficit—3 (10%) Language deficit—3 (10%) Vision deficit—1 (3%)	Motor deficit—3 (16%) Language deficit—1 (5%) Vision deficit—0 (0%)
Dayani, 2018	N—4/14 Four cases with immediate post-op deficits, one with persistent post-op deficits 1—Rt-sided hemiplegia, rt-sided neglect, slurred speech; slight improvement but persistent deficit 2—Blurred vision, seizure; improved to baseline levels 3—Lt-sided neglect, lt-sided weakness, apraxia; lost to FU 4—Expressive aphasia, perceptual motor difficulties, mild rt visual field cut; improved to baseline levels	NR
OpokuDarko, 2018	N—2/10 1—A significant thromboembolic complication resulting in multifocal infarcts in the peritumoral area and distal left ACA territory. Resulted in right hemiparesis, expressive dysphasia, and abulia 2—A postoperative epidural hemorrhage requiring evacuation and admission to the intensive care unit.	N—1/20 1—Postprocedural hemorrhage leading to obstructive hydrocephalus that required permanent placement of a ventriculoperitoneal shunt.
Boaro, 2019	N—10/26 with new post-operative deficits Motor deficit—5 Language deficit—3 Cognitive deficit—5 Other: Seizures—3 Post-op hydrocephalus—5 Deep Vein Thrombosis/Pulmonary Embolism—4	NR
Hall, 2019	7/36 with postoperative complications	5/36 with postoperative complications
Franco, 2020	N—8/25 Hemorrhage—3 Ischemia—1 Cranial nerve deficit—1 Meningitis—2 Hydrocephalus—1 Aphasia—1 Pneumonia—0	N—5/30 Hemorrhage—0 Ischemia—0 Cranial nerve deficit—0 Meningitis—0 Hydrocephalus—2 Aphasia—2 Pneumonia—1

NR: Not reported.

## Appendix D

**Table A2:** Quality assessment performed by authors using the questionnaire by Chan and Bhandari.

Study	Clear Study Objectives/Question	Well Defined Study Protocol	Explicit Inclusion/Exclusion Criteria for Participants	Specified Time Interval for Patient Recruitment	Consecutive Patient Enrolment	Clinically Relevant Outcomes	Prospective Outcome Data Collection	High Follow-Up Rate	Overall
Dziurzynski, 2012	1	1	1	1	1	1	0	1	7
Chaichana, 2014	1	1	1	1	0	1	0	1	6
Dayani, 2018	1	1	1	1	0	1	0	1	6
OpokuDarko, 2018	1	1	1	1	0	1	0	1	6
Boaro, 2019	1	1	1	1	0	1	0	1	6
Franco, 2020	1	1	1	1	0	1	0	1	6
